# Phase I/II study of immunotherapy using tumor antigen-pulsed dendritic cells in patients with hepatocellular carcinoma

**DOI:** 10.3892/ijo.2012.1626

**Published:** 2012-09-11

**Authors:** FUJIMASA TADA, MASANORI ABE, MASASHI HIROOKA, YOSHIOU IKEDA, YOICHI HIASA, YOON LEE, NAM-CHUL JUNG, WOO-BOK LEE, HYUN-SOO LEE, YONG-SOO BAE, MORIKAZU ONJI

**Affiliations:** 1 Department of Gastroenterology and Metabology, Ehime University Graduate School of Medicine, To-on, Ehime 791-0925, Japan;; 2 JW CreaGene Research Institute, JW CreaGene Inc., Sangdaewon-dong, Jungwon-gu, Seongnam-si, Gyeonggi-do 462-120;; 3 Department of Biological Sciences, Sungkyunkwan University, Jangan-Gu, Suwon, Gyeonggi-do 440-746, Republic of Korea

**Keywords:** dendritic cells, hepatocellular carcinoma, clinical trial

## Abstract

Dendritic cells (DCs) are increasingly used as adjuvants for vaccination strategies; however, there has been very little development in DC vaccines for patients with hepatocellular carcinoma (HCC). In this study, we assessed the safety, feasibility and efficacy of a multiple tumor-associated antigen (TAA)-pulsed DC vaccine in 5 patients with advanced HCC. DCs were generated by culturing blood monocytes in the presence of granulocyte macrophage-colony stimulating factor and interleukin-4 for 5 days. The DC vaccine was prepared by pulsing DCs with cytoplasmic transduction peptide-attached α-fetoprotein, glypican-3 and MAGE-1 recombinant fusion proteins and cultivating them in the presence of maturation cocktail. DCs were injected subcutaneously near the inguinal lymph nodes, followed by topical application of toll-like receptor-7 agonist around the injection site. We showed that our DC vaccine was safe and well-tolerated over 6 vaccinations in 5 patients. All 5 patients showed T cell responses against TAAs. Clinical benefit was observed in one of the 5 patients. In conclusion, the feasibility, safety and immune activity of DCs pulsed with TAAs were confirmed in HCC patients. However, clinical response was detected only in one patient. Future trials may consider applying this therapy in a less advanced stage to obtain better clinical responses.

## Introduction

Hepatocellular carcinoma (HCC) is one of the most prevalent malignant diseases worldwide (1). Many locoregional therapeutic approaches, including surgical resection, radio-frequency ablation (RFA), percutaneous ethanol injection (PEI), and transcatheter hepatic arterial chemoembolization (TACE) have been applied in the search for curative treatments for HCC. Although current advances in therapeutic modalities have improved the prognosis of patients with HCC, the survival rate is still unsatisfactory (2). One reason for the poor prognosis is the high rate of recurrence after treatment (3–5). Current therapeutic approaches do not prevent tumor recurrence efficiently.

Patients with HCC demonstrate some dysfunctions in their immune system, including abnormal innate and adaptive immune responses (6). Therefore, one strategy to reduce tumor recurrence is to enhance antitumor immune responses that may induce sufficient inhibitory effects to prevent tumor cell growth and survival. Dendritic cells (DCs) are professional antigen presenting cells that play a central role in the immune system by initiating an antigen-specific cytotoxic T lymphocyte (CTL) response (7,8). DCs acquire antigens through endocytosis and phagocytosis in peripheral tissues in their immature state and become mature. Subsequently, mature DCs migrate via blood and lymphatics to the secondary lymphoid organs, where they prime T cells. Due to their unique capacity to regulate T cell immunity, DCs are increasingly used as adjuvants for vaccination strategies. Recently, several studies have been performed using DC generated *ex vivo* from peripheral blood, and no significant toxicities were observed in the majority of patients. In addition, induction or enhancement of cellular immune responses against tumor antigens was found after DC vaccination (9,10).

Although immunotherapy strategies to eliminate HCC have consistently demonstrated high efficacy in animal models, very limited efficacy has been demonstrated in patients (11–20). There are possible explanations that may explain this discrepancy, but one major limitation for clinical trials is obtaining adequate amounts of immunogenic tumor-associated antigens (TAAs). DC loaded with autologous tumor or tumor lysates, which contain TAAs, are most frequently used for clinical trials (11–13,16). Another approach is to use apoptotic or necrotic tumor cells, which are induced by the standard treatments for HCC, as tumor antigens. Previous studies have shown that these cells effectively cross-prime the T cell response and induce potent immunity (14,15,18,20).However, ideal protocols to induce antigen-specific immunity involve DC loaded with TAAs themselves if such TAAs have been defined. Although many specific proteins have been identified with differential expression profiles in HCC cells, appropriate antigens for incorporation into DC vaccines for HCC have not been defined. α-fetoprotein (AFP) is a potential candidate antigen, and Butterfield *et al* reported that DC pulsed with HLA-A0201-restricted peptides induced AFP-specific T cell responses, though no clinical responses were observed (17).

To overcome these problems, we conducted a phase I/II clinical study using DC vaccine prepared as follows: i) TAA-pulsed mature DCs were used together with topical application of toll-like receptor (TLR)-7 agonist; ii) recombinant proteins, instead of epitope peptides, were used as a source of TAA to overcome the restriction of HLA type; iii) 3 different HCC antigens were used to cover the broad spectrum of HCC heterogeneity; iv) for efficient delivery of antigens into the cytoplasm of DC, cytoplasmic transduction protein (CTP)-mediated transduction system (21) was used. The primary objective of this study was to assess the safety, feasibility and immune activity of multiple TAA-pulsed DC therapy. The efficacy of this therapy was also evaluated.

## Patients and methods

### 

#### Patient selection

The clinical trial protocol was approved by the Institutional Review Board of Ehime University Hospital (Approval ID #0809003). Patients were informed of the investigative nature of this study, and written consent in accordance with institutional regulations was obtained prior to study entry. Eligibility criteria included radiological diagnosis of primary HCC by computed tomography (CT), classified in stage II and III according to the tumor-node-metastasis (TNM) classification; age over 20 years/both male and female; Eastern Cooperative Oncology Group scale 0–1; and indicatiors of acceptable hematological (hemoglobin ≥8.5 g/dl, white blood cells ≥2,000/mm^3^, platelet ≥50,000/mm^3^), hepatic (Child Pugh score ≤7, alanine aminotransferase, aspartate aminotransferase ≤5x upper normal limit) and renal (creatinine ≤1.5 mg/dl) function. Important exclusion criteria consisted of organ transplantation; a medical history of autoimmune disease, immunodeficiency, or autoimmune disease that might be aggravated by immunotherapy; not exceeding 2 weeks after antibiotic treatment needed due to a serious infectious disease; seropositivity for human immunodeficiency virus antigen; use of immunosuppressive drug such as cyclosporin A and azathioprine; any cardiopulmonary disability judged by the investigator; a medical history of psychological disease or epilepsy; and evidence of another active malignant neoplasm.

#### Preparation of recombinant hepatocellular carcinoma antigens

cDNAs encoding AFP, MAGE-1 or glypican-3 (GPC3) were cloned into the pCTP vector (21). These 3 antigens were expressed in the form of 6x-His-attached fusion proteins in *E. coli* BL21 (DE3) and purified using nickel-nitrilotriacetic acid (Ni-NTA) column chromatography (Qiagen, Hilden, Germany). The recombinant antigen production and purification were performed at Good Manufacturing Practice (GMP)-compliant facility following the Korean Food and Drug Administration (KFDA) guideline. Each antigen was certified through the process of quality control: purity >95% in SDS-PAGE analysis and endotoxin <1.0 EU/*μ*g in Limulus amebocyte lysate test.

#### Autologous DC generation

DCs were generated from blood monocytes, as reported previously (22), with modifications. White blood cells obtained from the HCC patients through leukapheresis. DCs were prepared in a GMP-compliant facility at Ehime University Hospital (Ehime, Japan). Peripheral blood mononuclear cells (PBMCs) were separated from WBC by Ficoll-Paque™ PLUS (Amersham Biosciences, Uppsala, Sweden) density gradient centrifugation. PBMCs were stored in a liquid nitrogen tank until necessary for DC generation. PBMCs thawed, washed with Hanks’ Balanced Salt Solutions, resuspended in RPMI-1640 medium (Lonza, Basel, Switzerland) supplemented with autologous heat-inactivated plasma, and then incubated in CellSTACK Culture Chambers (Corning, Corning, NY, USA). After 0.5–1 h incubation at 37°C in a 5% CO_2_ incubator, non-adherent cells were removed by gentle washes.

The adherent monocytes were cultured in X-VIVO15 (Cambrex, East Rutherford, NJ, USA) supplemented with 100 ng/ml of granulocyte macrophage-colony stimulating factor (GMP grade: LG Life Science, Seoul, Korea) and 300 ng/ml of interleukin (IL)-4 (JW CreaGene Inc., Seongnam, Korea) for 5 days. On day 5, nonattached immature DCs were harvested and pulsed with CTP-fused human AFP, MAGE-1 and GPC-3 recombinant proteins at a final concentration of 5 *μ*g/ml each. Antigen-pulsed dendritic cells were matured in the presence of cytokine cocktail, IL-6 (Peprotech, Rocky Hill, NJ, USA), IL-1β (Peprotech), tumor necrosis factor (TNF)-α (Peprotech), prostaglandin E_2_ (PGE2) (Sigma Chemical Co., St. Louis, MO, USA), interferon (IFN)-γ (LG Life Science), OK432 (Chugai Pharmaceutical Co., Tokyo, Japan), and poly I:C (Sigma) for 1 or 2 days depending on surface phenotypes and cell population. On day 6–7, the DCs were harvested, washed, and resuspended in 1.2 ml of cryopreserving solution containing 5% dimethyl sulfoxide (Bioniche Pharma USA, Lake Forest, IL, USA). Finally fully equipped DCs were packed into a sterile glass vial (4×10^7^ cells/vial), sealed with a snap-cap, and stored at an ultralow freezer for >12 h.

### Quality control of dendritic cell vaccine

#### Safety test

For safety, endotoxin, germ-free and mycoplasma-free tests were performed according to the KFDA-approved JW CreaGene standard and test guidelines. Endotoxin was evaluated using gel-clot techniques. The endotoxin of the product should be less than 10 EU/ml per 1.2-ml vial. Mycoplasma test was performed by both direct culture and PCR methods using e-Myco™ Mycoplasma PCR detection kit (Intron Biotechnology, Seongnam, Korea), which contains primer sets specifically designed to detect major contaminants of *Mycoplasma* in cell cultures such as *M. arginini*, *M. faucium*, *M. fermentans*, *M. hyorhinis*, *M. orale*, and *A. laidlawii* as well as other broad spectrum of mycoplasma.

#### Cell size and granularity

During the differentiation from monocytes to dendritic cells, cell size and granularity increase. Based on these principles, the cell size and granularity of each DC vaccine were assessed by flow cytometric analysis. PBMCs were used for gating control.

#### Phenotypic analysis

The phenotype of DC vaccine was determined by flow cytometry using a FACSCalibur™ flow cytometer (Becton Dickinson, Franklin Lakes, NJ, USA). The following monoclonal antibodies were used: i) fluorescein isothiocyanate-conjugated mouse antihuman IgG2a isotype control; ii) phycoerythrin-conjugated mouse antihuman IgG1 isotype control; iii) anti-CD14, anti-CD19, anti-CD40, anti-CD80, anti-D86, anti-HLA-ABC, and anti-HLA-DR (BD Pharmingen, San Diego, CA, USA).

#### Viability

The viability of DC vaccine was assessed by propidium iodide (PI) staining. PI (BD Pharmingen) was added to a sample and kept in the dark at room temperature for 20 min. Cell viability was examined by flow cytometry using a FACSCalibur™ (Becton Dickinson). Viability was represented as 100-[(PI^+^ of sample)−(PI^+^ of control)] (%).

#### Lymphocyte proliferation assay

One vial from each DC vaccine lot was used to test of T cell stimulation capacity according to the standard lymphocyte proliferation assay. T cells were isolated from cryopreserved PBMC using nylon wool column (Polysciences, Warrington, PA, USA). Purified T cells (1×10^5^) were cultured with serially diluted DC vaccine (starting from 1×10^4^ cells to 0.33×10^3^ cells) at 37°C for 5 days. T cell proliferation was assessed by 3-(4,5-di-methylthiazol-2-yl)-2,5-diphenyltetrazolium bromide, yellow tetrazole: MTT) assay following manufacturer’s protocol (CellTiter 96 Non-Radioactive proliferation assay kit; Promega, Madison, WI, USA). R2 represent the standard curve of MTT assay for the validation of a data set.

#### Cytokine production assay

Either culture supernatant of each antigen-pulsed DC or co-cultured medium of T cells/DC at the ratio of 10:1 was collected and stored at −80°C until this assay. The concentration of IL-12p70, IL-10, IFN-γ, and IL-4 was measured with corresponding human immunoassay kits (BD OptEIA^™^ kit, BD Pharmingen) based on the manufacturer’s instruction. Each experiment was performed 3 times and the result was described as the mean ± standard deviation.

#### Treatment protocol (Fig. 1)

The screening evaluation was performed 3 weeks before the start of immunotherapy and consisted of the following: complete history, thorough physical examination, chest X-ray, electrocardiogram, urine analysis, hematological and immunological parameters, serum chemistry, tumor markers [AFP and protein induced by vitamin K absence or antagonists-II (PIVKA-II)], ultrasonography and abdominal CT scan. Eligible patients underwent TACE 2 weeks before the start of the vaccination. PBMC collection by leukapheresis was performed 1 week before the first planned vaccination. Tumor antigen-pulsed DCs were injected subcutaneously into the thigh near the inguinal lymph nodes. Topical TLR-7 agonist (imiquimod; Aldara ^™^ Cream; Mochida Pharmaceutical Co., Tokyo, Japan) applied around the injection site from 2 consecutive days before injection. During the first cycle, 4 vaccinations were administered at biweekly intervals. Medical history and standard blood tests and urine analysis were performed at each vaccination. Vital signs were monitored during and after each injection. Response evaluation was performed 4 weeks after fourth vaccination (10 weeks after first vaccination), and TACE was repeated. Two further vaccinations were administered at biweekly intervals, and final response evaluation was performed at 18 weeks after first vaccination. Tumor markers and serological tests for autoantibodies, including anti-nuclear antibody, were evaluated every 4 weeks.

#### Clinical response and toxicity assessment

Clinical responses to vaccination were evaluated according to the Response Evaluation Criteria in Solid Tumors (RECIST) criteria (23). Complete response was defined as disappearance of all target lesions. Partial response was defined as 30% decrease in the sum of the longest diameter of target lesions. Progressive disease was 20% increase in the sum of the longest diameter of target lesions. Stable disease was defined as small changes that do not meet above criteria. Toxities were classified according to the National Cancer Institute Common Toxicity Criteria.

#### Analysis of IFN-γ-producing cells using enzyme-linked immunospot (ELISPOT) assay

The ELISPOT assay was adopted to detect and enumerate individual cells that secrete IFN-γ *in vitro* upon HCC-specific or -associated tumor antigens. Human IFN-γ ELISPOT pair antibodies were purchased from BD Pharmingen, and ELISPOT assay was performed according to the manufacturer’s instruction. In brief, PBMC (2×10^5^ cells) treated with each antigen (3–5 *μ*g/ml) or antigen mixtures were loaded on a flat-bottomed 96-well ELISPOT plate (Millipore, Danvers, MA, USA) precoated with capture antibody. The plate was incubated for 20 h at 37°C CO_2_ incubator. After washing, detection antibody was added to each well and incubated for 2 h at room temperature. Avidinhorseradish peroxidase conjugate was added to each well, and the plate was developed with 3-amino-9-ethyl-carbazole substrate reagent set. Visible spots were enumerated using an automated ELISPOT reader (CTL, USA) and default program.

## Results

### 

#### Patients

Treatment was performed at Ehime University, in 2009 (Ehime, Japan). Baseline characteristic of the 5 patients enrolled are shown in Table I. The basis of the diagnosis of HCC was histological and/or radiolgical for all patients. All patients were male with age range 46–64 years. Two and 3 patients were infected with hepatitis B virus and hepatitis C virus, respectively. All patients were previously treated with TACE.

#### DC vaccine

**Figure 2.**
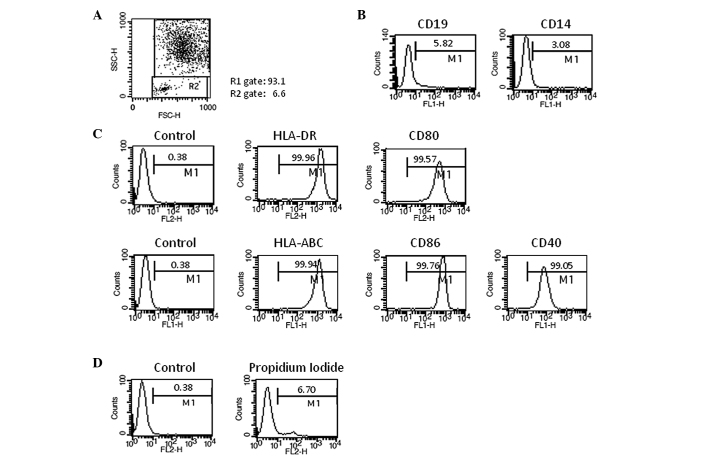
Quality control of DC vaccine. DC vaccines generated in the institutional GMP facility were analyzed by FACS. (A) Cell size (FSC) and granularity (SSC) were analyzed in FACS light scattered plots. (B) Purity was assessed by measuring the contamination of CD14^+^ and CD19^+^ cells among DC gated cells. (C) The expression of MHC class I (HLA-ABC), MHC class II (HLA-DR), and costimulatory molecules such as CD80, CD86, and CD40 was assessed by FACS. The percentages of positive cells are indicated. (D) DC viability was assessed by propidium iodide exclusion methods.

DC vaccine was generated successfully from the 5 patients with HCC. One vial from each lot of frozen DC vaccines was thawed and used for quality control. DC vaccine demonstrated typical features of mature DC morphology under a microscope. The cell population in DC gate in the FACS analysis was over 90% on the basis of the cell size and granularity, with a median value of 94.4% (Fig. 2A). The analysis of lineage markers revealed that the contamination of B cells (CD19) and monocytes (CD14) was less than 10% (Fig. 2B). Over 95% of DCs demonstrated MHC class I (HLA-ABC) high, MHC class II (HLA-DR) high, and costimulatory molecules (CD86, CD80, and CD40) high (Fig. 2C). These characteristics were commonly maintained in all 5 different DC vaccines, which were generated under the same culture conditions. Viability is one of the most important issues in DC vaccine. The viabilities of DC vaccines ranged from 86.2% to 93.5% and median value was 92.3% (Fig. 2D), indicating that the frozen DCs can be used as a therapeutic vaccine. The frozen DC vaccine was stable for longer than 6 months (data not shown). The purity, cell viability and surface phenotypes of 5 different DC vaccines are summarized in Table II.

#### Cytokine production assay

To determine whether DC vaccine was functionally active to induce Th1 immune responses, we examined IL-12 and IL-10 production from DC induced by each specific antigen such as AFP, GPC-3, or MAGE-1. As a result, IL-12 was highly produced whereas the amount of IL-10 production was almost a basal level (Table IIIA). Furthermore, predominant IFN-γ level in T cell/DC co-cultured supernatant from those five HCC patients was also confirmed, while the level of IL-4 production was <15 pg/ml (Table IIIB).

#### Toxicity assignment

Injection of DC vaccine was safe and well tolerated. Toxicity was mild and no grade III/IV serious adverse events occurred in a total of 30 instances of cell injection (Table IV). No hematological, hepatic or renal toxicities or *de novo* autoantibody formation were observed in any patient.

#### Clinical response assessment

**Figure 3.**
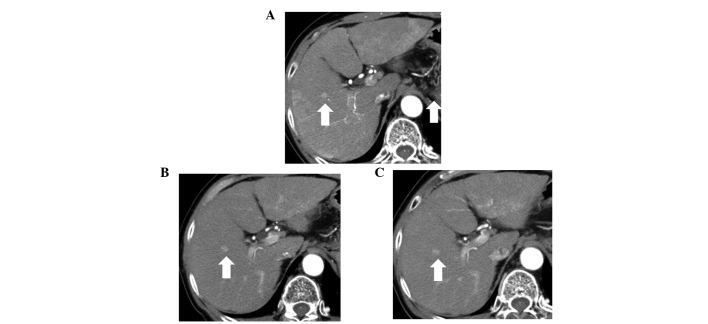
Clinical response to DC vaccination. Dynamic CT scans (arterial phase) of patient no. 3. (A) Before vaccination. (B) Four weeks after fourth vaccination (10 weeks after first vaccination). (C) Four weeks after final (sixth) vaccination. Arrow indicates the target lesion.

One patient (patient no. 3) achieved disease stabilization during the follow-up period (Fig. 3), however, no tumor response was observed in the other 4 patients (Table I). Serum AFP levels decreased in 2 patients; however, serum PIVKA-II levels increased in all patients.

#### T cell responses after DC vaccination

**Figure 4.**
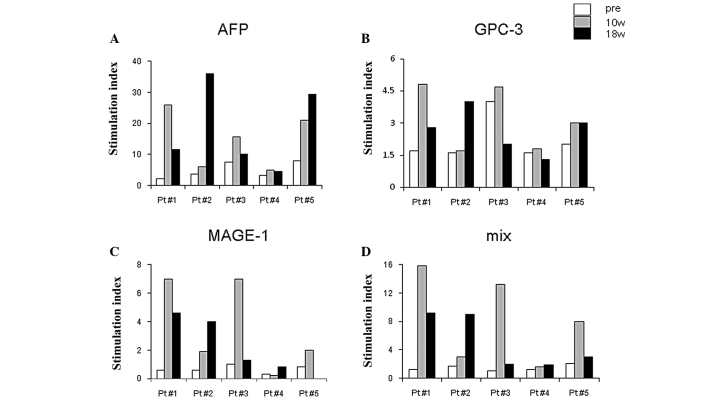
IFN-γ ELISPOT assays after DC vaccination. The PBMCs obtained from 5 patients after DC vaccinations were assessed by ELISPOT assay. PBMCs were incubated with or without each soluble HCC antigen (5 *μ*g/ml) (A–C) or antigen mixture (D) for 24 h in 96-well ELISPOT plates, and the IFN-γ + spots were assessed by ELISPOT reader. Data show the number of IFN-γ + spots per 2×10^5^ PBMCs. Pre: before vaccination, 10w: 4 weeks after fourth vaccination, 18w: 4 weeks after final (sixth) vaccination.

After DC vaccination, all 5 patients demonstrated strong T cell responses against HCC antigens compared with the samples obtained before vaccination. The stimulation index (SI) shown in Fig. 4 illustrates the high reactivity of AFP antigen in all 5 patients after vaccination, while GPC-3 or MAGE-1 antigens were moderate in their capacity to induce T cell responses. AFP-specific IFN-γ-producing cells peaked 10 weeks after the first vaccination in 2 patients, and 18 weeks in 2 patients.

## Discussion

HCC is one of the major malignancies in Asian countries including China, Korea and Japan (1). Screenings based on imaging studies, such as ultrasonography and CT, and serum tumor markers have improved HCC detection in high-risk patients at a relatively early stage. Such patients may have some benefits by curative treatments for inhibition of local recurrence in the liver; however, the surrounding non-tumor liver tissues exhibit a high carcinogenic potential, such as liver cirrhosis and chronic hepatitis. The high rate of intrahepatic recurrence is a key feature correlated with poor prognosis, and its prevention is an issue for urgent investigation (5).

HCC is a potentially ideal tumor for targeting by immune-based therapies (24–26). However, the observation of tumor progress in HCC despite the presence of tumor-specific immune responses suggests that development of HCC leads to a number of immune suppressor mechanisms, including increase of regulatory T cells (27), myeloid-derived suppressor cells (28), and impairment of antigen-presenting cells. DCs are the most potent antigen-presenting cells effective to induce appropriate adaptive immune responses (7,8). However, DC function is suppressed in patients with HCC (29,30), and may lead to a failure of the induction and maintenance of antitumor immunity. Therefore, these observations provide a rationale for activating DC *in vitro* and infusing them into patients to overcome tumor-related immunosuppression to induce sufficient anti-tumor immunity. A series of clinical trials using DC-based vaccines demonstrated evidence of safety and immune activity; however, clinical benefits have shown to be limited (11–20). Therefore, clinical trials with a well established DC vaccination protocol are highly recommended in the field of DC-based immunotherapy.

We investigated the safety and efficacy of the autologous DC-based tumor vaccine charged with HCC-specific/associated recombinant antigens in 5 patients with advanced HCC. No technical hardships were encountered with blood procurement or the subsequent generation of DC vaccine. No severe treatment-related complications were noted (Table IV), and antigen-specific immunity was induced in all patients (Fig. 4). A clinical response, defined as stable disease (SD) was achieved in one patient (Fig. 3). These results indicate that DC vaccine used in this study is well tolerated and able to induce anti-tumor immunity in patients with HCC that may be associated with clinical benefits.

Our DC vaccine protocol for the treatment of the patients with HCC comprises major modifications from the previous studies in several points. First, we used mature DCs which were antigen-charged and stimulated with a cytokine mixture, poly I:C, and OK432 (Fig. 2 and Table II). Immature DCs have been used in several clinical trials (11–18). Evidence suggests that mature DCs are better in inducing clinical impact in DC-based cancer immunotherapy (31). Recently, Nakamoto *et al*(20) demonstrated that infusion of mature DCs, but not immature DCs, during the TACE procedures prolonged recurrence-free survival. Antigen uptake assay was not exactly preceded because of shortage of PBMC. However, based on another set of experiments which were performed using DC derived from HCC patients, the result of antigen uptake capacity of DC vaccine was always >70% evaluated by FITC-dextran uptake assay (data not shown). Second, topical application of imiquimod, a TLR7 ligand, was also used to enhance anti-tumor immunity in synergy with DC vaccine (32). Aldara™ Cream (5% imiquimod) is a new type of treatment in the category of medicines known as immune response modifiers and is indicated for the treatment of condyloma acuminate. In this study, we demonstrated the feasibility and safety of DC vaccine designed to have synergistic effects with imiquimod in HCC patients. Third, we used a novel approach for the delivery of tumor antigens into DCs. CTP has a strong membrane transduction potential (21), and was very efficient for the delivery of antigens into the cytoplasm of DCs. DC vaccine pulsed with CTP-conjugated antigens elicited a robust Th1-mediated immunity and antigen-specific CTL responses when compared with antigen alone, which is probably attributable to the CTP technology. The feasibility was confirmed in this clinical trial. Finally, we used 3 different recombinant proteins as a source of HCC antigens for the generation of DC vaccine. Because any single antigen is ubiquitously expressed in HCC, we selected AFP, GPC-3 and MAGE-1 as target antigens for DC vaccine through the analysis of the tissue array of a tumor tissues obtained from 412 patients with HCC in Korea (data not shown). AFP has been studied as a possible candidate antigen for anti-HCC immunotherapy. T-cell responses to AFP-CTL epitope peptides were strongly induced in patients with HCC (33,34). In addition, the overexpression of GPC-3 specifically in human HCC has been reported (35), and DC expressing GPC-3 induced protective immunity against highly meta-static cancer (36). Furthermore, the MAGE-1 was reported to be expressed in 30% to 78% in HCC tissue samples (37,38). An advantage of this approach is that recombinant proteins were used for equipping DC vaccine to overcome the HLA restriction of epitope peptides. In this study, AFP-specific T cell response was significantly induced in all 5 patients after DC vaccination, but those against GPC-3 and MAGE-1 were moderate even after DC vaccination (Fig. 4). Moderate responses to GPC-3 and MAGE-1 in the vaccine remain to be further characterized, but are likely, at least in part, attributable to the limited immunogenicity of each antigens *in vivo*. The recombinant protein CTP-GPC-3 does not have trans-membrane and cytoplasmic domains, latter of which contains immunogenic epitopes (39).

We could not investigate the expression pattern of each tumor antigen in HCC nodules for the limitations of biopsy samples. Therefore, we were not able to analyze correlation between TAA expression and TAA-specific T cell response after vaccination. Further studies are necessary in this regard. However, the results of the present study confirmed the feasibility, safety and immune activity of recombinant tumor antigen-pulsed DC vaccine for therapeutic use in HCC patients. Genome profiling studies of HCC have revealed that HCC is a very heterogeneous tumor (40). Furthermore, HCC demonstrates multicentric carcinogenesis and develops at different time points. These data indicate that the identification of many more target antigens and their optimization is necessary to evoke better clinical responses.

In conclusion, we conducted a phase I/II clinical trial using DC vaccine in 5 patients with advanced HCC and liver cirrhosis. DC vaccine was well tolerated in all patients and induced anti-tumor immune responses in vaccine, but clinical response was detected only in 1 patient (patient 3) with advanced HCC and liver cirrhosis. The tumor-load of this patient was relatively smaller compared to those of other 4 patients (Table I). Including our study, most of DC-based immunotherapies have been studied in patients with advanced stage disease, resulting in poor clinical responses. Future trials in less advanced disease may accompany better clinical responses. DC-based tumor immunotherapy will be a good indication as an adjuvant setting to radical therapy, such as surgical resection or RFA, to prevent tumor recurrences in patients with HCC.

## Figures and Tables

**Figure 1.**
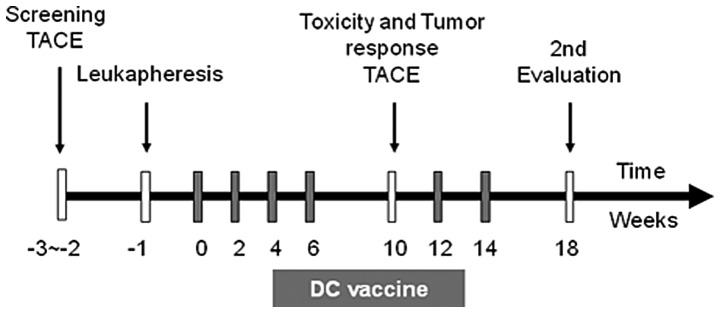
Study design and vaccination schedule. TACE, transcatheter hepatic arterial chemoembolization; DC, dendritic cells.

**Table I.** Patient characteristics and treatments.

A							
Patient no.	Sex	Age (years)	Etiology	TNM stage	No. of tumors	Largest tumor	Child-Pugh
1	M	65	HCV	III	2	22.1	A
2	M	58	HBV	III	2	15.9	A
3	M	59	HCV	II	1	6.6	A
4	M	64	HBV	II	1	12.4	A
5	M	46	HCV	II	9	30.3	B

B						
		AFP (<ng/m)		PIVKA-II (mAU/ml)		Outcome
Patient no.	Previous treatment	Pre	Post	Pre	Post	
1	TACE	23.7	56.7	842	3,109	PD
2	TACE	12.4	16.8	42	1,189	PD
3	TACE	27.4	23.2	53	67	SD
4	TACE	30.5	181.4	59	192	PD
5	TACE	854.1	660.0	10,707	30,615	PD

HBV, hepatitis B virus; HCV, hepatitis C virus; TACE, transcatheter hepatic arterial chemoembolization; AFP, α-fetoprotein; PIVKA-II, protein induced by vitamin K absence or antagonists-II; PD, progressive disease; SD, stable disease.

**Table II.** Quality control results of 5 different DC vaccines.

Patient no.	No. 1	No. 2	No.3	No. 4	No. 5
Sterility					
I	Pass	Pass	Pass	Pass	Pass
II	Pass	Pass	Pass	Pass	Pass
Mycoplasma					
I (PCR)	Pass	Pass	Pass	Pass	Pass
II (Direct culture)	Pass	Pass	Pass	Pass	Pass
Endotoxin (<10 EU/ml)	Pass	Pass	Pass	Pass	Pass
Viability (%)	86.2	91.2	92.7	93.5	**92.3**^a^
Identification					
Size & granularity (%)	93.7	94.8	**94.4**	97.3	90.0
Cell surface phenotypes (%)					
HLA-DR	99.8	99.0	98.9	99.7	**99.7**
HLA-ABC	99.5	99.8	99.9	**99.8**	99.9
CD86	**99.6**	98.9	99.4	99.9	99.8
CD80	95.6	98.9	98.9	99.4	**99.1**
CD40	87.9	**97.2**	98.9	95.0	97.9
Purity test					
CD14	8.5	7.1	3.1	3.5	2.1
CD19	0.9	0.6	1.6	0.8	1.3
Total cell number (×10^7^)	4.1	4.08	4.24	4.22	4.25
T cell proliferation					
DC 1×10^4^ cells	Not tested	Not tested	0.777	0.849	0.908
DC 0.33×10^3^ cells			0.349	0.439	0.343
Coefficient factor (R^2^)^*^			0.989	0.948	0.993

a Bold letter represents median value of each test set.

**Table III.** Cytokine production assay results of 5 different DC vaccines.

A			

Patient no.	Antigens	IL-12p70 (ng/ml)	IL-10 (ng/ml)
1	AFP	35.3±3.5	0.13±0.03
	GPC-3	32.3±3.0	0.014±0.002
	MAGE-1	58.8±3.0	0.65±0.15
2	AFP	3.3±0.5	0.04±0.01
	GPC-3	5.5±0.6	0.05±0.01
	MAGE-1	31.1±4.9	0.34±0.15
3	AFP	9.0±0.8	0.013±0.006
	GPC-3	13.4±1.0	0.04±0.01
	MAGE-1	43.9±4.4	0.23±0.06
4	AFP	1.9±0.3	0.09±0.02
	GPC-3	2.0±0.4	0.09±0.04
	MAGE-1	11.0±1.4	0.37±0.07
5	AFP	3.1±0.5	0.53±0.06
	GPC-3	2.6±0.6	0.07±0.01
	MAGE-1	2.3±0.4	0.08±0.05

B	

Patient no.	IFN-γ (ng/ml)
1	16.5±0.9
2	10.4±2.9
3	20.5±3.3
4	9.3±0.8
5	18.9±2.3
Positive control	23.5±3.3
Negative control	0.1±0.04

(A) IL-12 and IL-10 production in DC culture supernatant which was derived from 5 individual HCC patients. The amount of cytokine production induced by each specific antigen was measured. (B) Cytokine levels in T cell/DC co-cultured supernatant from 5 HCC patients. Positive control was from T cell/keyhole limpet hemocyanin (KLH)-pulsed DC co-culture supernatant, and the negative control was from the supernatant which was cultured with T cell alone. Each experiment was performed 3 times and the result was described as the mean ± standard deviation (n=3).

**Table IV.** Toxicity profiles by patients.

Toxities	Grade 1	Grade 2	Grade 3	Grade 4
Injection site reaction	5/5	-	-	-
Fever	4/5	1/5	-	-
